# Naphtha in Phthisis

**Published:** 1844-06-29

**Authors:** 


Naphtha in Phthisis.—Dr. Powell gives a brief note of three cases of phthisis in which he gave a trial to the internal exhibition of purified naphtha, in two of which it was not of any service, and in the third it was decidedly prejudicial.

